# Genome-wide analysis for the melatonin trait associated genes and SNPs in dairy goat (*Capra hircus*) as the molecular breeding markers

**DOI:** 10.3389/fgene.2023.1118367

**Published:** 2023-03-20

**Authors:** Hao Wu, Qi Yi, Wenkui Ma, Laiqing Yan, Shengyu Guan, Likai Wang, Guang Yang, Xinxing Tan, Pengyun Ji, Guoshi Liu

**Affiliations:** ^1^ National Engineering Laboratory for Animal Breeding, Key Laboratory of Animal Genetics and Breeding of the Ministry of Agricultural, Beijing Key Laboratory for Animal Genetic Improvement, College of Animal Science and Technology, China Agricultural University, Beijing, China; ^2^ Sanya Institute of China Agricultural University, Sanya, China; ^3^ Hainan Yazhou Bay Seed Laboratory, Sanya, China; ^4^ Inner Mongolia Grassland Hongbao Food Co., Ltd., Bayannaoer, China

**Keywords:** dairy goat, melatonin, BSA analysis, AANAT, ASMT, SNP

## Abstract

Previous studies have reported that the endogenous melatonin level is positively associated with the quality and yield of milk of cows. In the current study, a total of 34,921 SNPs involving 1,177 genes were identified in dairy goats by using the whole genome resequencing bulked segregant analysis (BSA) analysis. These SNPs have been used to match the melatonin levels of the dairy goats. Among them, 3 SNPs has been identified to significantly correlate with melatonin levels. These 3 SNPs include CC genotype 147316, GG genotype 147379 and CC genotype 1389193 which all locate in the exon regions of ASMT and MT2 genes. Dairy goats with these SNPs have approximately 5-fold-higher melatonin levels in milk and serum than the average melatonin level detected in the current goat population. If the melatonin level impacts the milk production in goats as in cows, the results strongly suggest that these 3 SNPs can serve as the molecular markers to select the goats having the improved milk quality and yield. This is a goal of our future study.

## Introduction

Dairy sheep and goat account for about 21% of all sheep species and most of them are distributed in subtropical areas of Asia, Europe, and Africa (Pulina et al., 2018). The goat milk is used to make a variety of dairy products including butter, ice cream, cheese, buttermilk, yogurt, flavored milk and they are the popular human diet (Nayik et al., 2021). Goat milk characterizes with relatively higher milk fat than cow milk and it is nutritional value is very similar to human milk; therefore, it is considered importance for human health including prevention of cardiovascular diseases, cancer, allergies, and microorganism infection (García et al., 2014; Clark and Mora Garcia, 2017). As a result, many countries are expanding their dairy goat industry. It was estimated that the global production of goat milk was around 18.7 million tons in 2017, an increase of 16% compared to the 2007 (Miller and Lu, 2019). In addition, more and more modern technologies of molecular biology have been used to increase the dairy goat breeding and improve the milk quality as well as the yield. In the previous study, we have found that melatonin supplementation significantly reduces the milk somatic cell number and increases the milk protein and fat contents in cows (Wu et al., 2021). This observation makes us to think whether the enhanced endogenous melatonin production also has the similar result.

Melatonin was believed to be mainly produced by the pineal gland in vertebrates (Amaral and Cipolla-Neto, 2018). Currently, melatonin has been identified to be synthesized in other tissues and organs. These include gastrointestinal tract, brain, liver, kidney, adrenal gland, heart, thymus, gonad, placenta and uterus (Acuna-Castroviejo et al., 2014). Melatonin is produced in the mitochondria and thus, virtually, all cells having mitochondria can synthesize melatonin (Reiter et al., 2017). Melatonin is a potent antioxidant, anti-inflammatory and immunoregulatory molecule. It also participates in the regulatory activities in central nerve, immune, respiratory, digestive and urinary systems (Singh and Jadhav, 2014). In mammals, melatonin synthesis is under the control of gene expression of both aralkylamine N-acetyltransferase (AANAT) and acetylserotonin O-methyltransferase (ASMT) which are the rate-limiting enzymes for melatonin synthesis while some of its biological functions are mediated by its membrane receptors 1 and 2 (MT1 and MT2), respectively (Wang et al., 2014). Even though the melatonin synthetic pathway has been well documented, its fine regulatory mechanisms especially in the level of single nucleotide polymorphism (SNP), have not been extensively studied.

SNP mainly refers to DNA sequence polymorphism at the genome level, which is the most ideal marker for DNA inheritance and molecular breeding (Kruglyak, 1997). It can be used to establish a variety of analytic models to estimate the degree of heritable variation and phenotypes, thus, it can be used as the biomarker for biological breeding process (Tang et al., 2022). Yao et al. (2022) have found the key SNPs in genes of melatonin synthesis in Holstein dairy cows. Whether these similar SNPs existing in goats are unknown. Currently, the multiple SNP chips for dairy and cashmere goat are available, and the genome-wide analysis for some important traits of goats has been conducted by several researchers (Mucha et al., 2018; Jin et al., 2020). In addition, the SNP in the whole genome related to the selected traits in general populations can be quickly identified by the BSA method (Li et al., 2022). This method is widely used in molecular marker screening and quantitative trait locus (QTL) mapping analysis to target traits in plants and animals (Kurlovs et al., 2019; Li and Xu, 2022). BSA has been successfully used to analyze the F2 and F3 populations of the crossover between Wagyu and Qinchuan cattle and has identified four potential SNPs related to intramuscular fat traits (Zhu et al., 2021).

In this study, BSA method will be used to analyze the genotypes of individual dairy goats to match up their endogenous melatonin levels. The focus will be given to the SNP polymorphisms of AANAT/ASMT as well as MT1/MT2. Our purpose is to identify whether SNPs among these genes are associated with melatonin production in dairy goats. If this is the case, the SNPs can be used as molecular markers to select dairy goats with the naturally high melatonin production.

## Materials and methods

### Sample collection

A total of 103 healthy, 18-month-old dairy goats in their peak lactation period were selected. 5 mL of blood were collected from subcutaneous vein at the neck, centrifuged for 8 min at 3,000 r/min, and serum was collected and stored at −20°C for future use. The breast was cleaned with wash, and 5 mL of milk sample was collected and stored at −20°C for future use.

### Melatonin assay

Melatonin was dissolved in methanol (chromatographic grade) to make 1 mg/mL melatonin stock solution. The stock solution was successively diluted into 100, 50, 20, 10, and 5 ng/L to set up the melatonin test standard curve. 200 μL of blood or milk were mixed into 800 μL of cold methanol, vortexed for 30 min, centrifuged at 14,000 r/min at 4°C for 20 min. The supernatant was filtered with a filter size of 0.22 μm and stored in a brown sample bottle at −20°C for future use. Melatonin was detected using Liquid chromatography tandem mass spectrometry (LC-MS/MS) (Santa Clara, CA, United States) in the Central Laboratory of Beijing Institute of Animal Science, Chinese Academy of Sciences. The C18 column was used to separate the melatonin in the samples.

### DNA re-sequencing by Illumina HiSeq

Goat blood DNA was extracted following the instructions of the manufacturer’s protocol (NEBNext^®^ Ultra™ DNA Library Prep Kit for Illumina^®^). For each sample, 1 μg genomic DNA was randomly fragmented to <500 bp by sonication (Covaris S220). The fragments were treated with End Prep Enzyme Mix for end repairing, 5′ Phosphorylationand dA-tailing in one reaction, followed by aT-A ligation to add adaptors to both ends. Size selection of Adaptor-ligated DNA was then performed using AxyPrep Mag PCR Clean-up (Axygen) and fragments of ∼410 bp were recovered. Each sample was then amplified by PCR for eight cycles using P5 and P7 primers, with both primers carrying sequences which can anneal with flow cell to perform bridge PCR and P7 primer carrying a six-base index allowing for multiplexing. The PCR products were cleaned up using AxyPrep Mag PCR clean-up (Axygen), validated using an agilent 2,100 bioanalyzer (agilent technologies, PaloAlto, CA, United States) and quantified by Qubit2.0 fluorometer (invitrogen, carlsbad, CA, United States)

Then, libraries with different indexes were multiplexed and loaded on an Illumina HiSeq instrument according to manufacturer’s instructions (Illumina, San Diego, CA, United States). Sequencing was carried out using a 2 × 150 paired-end (PE) configuration; image analysis and base calling were conducted by the HiSeq Control Software (HCS) + OLB + GAPipeline-1.6 (Illumina) on the HiSeq instrument.

### Raw data quality control analysis

For the original image data of sequencing results, the software BCF2FASTQ (version 2.17.1.14) was used for Base calling and preliminary quality analysis to obtain the original Data of sequencing samples (PF), which is stored in FASTQ (FQ) file format. The data of Pair End sequencing consists of two FQ files, one storing read segment 1 (Read1) and the other storing read segment 2 (Read2). Cutadapt (version 1.9.1), the second-generation sequencing Data quality statistics software, was used to remove connectors and low-quality sequences from the original sequencing Data (Pass Filter Data) to obtain Clean Data for subsequent information analysis. Using Dragen Genome Pipeline, Clean data were aligned to the reference Genome sequence, and the resulting BAM (binary SAM file) was compared. Picard and GATK were used for PCR duplicate removal, local realignment, and base quality recalibration. The corrected genome alignment results were obtained. The coverage and coverage depth of the genome were calculated according to the comparison results.

### Full SNP detection and annotation

Variant detection was performed using the Haplotype Caller module of the software GATK (version 4.0.4.0) based on the comparison results of Clean Reads in the reference genome. Filtering was performed using the Variant Filtration module with the filtering parameter: -filter expression “QD < 2.0 || MQ < 40.0 || FS > 60.0 || SOR >3.0 || MQ Rank Sum < −12.5 || Read Pos Rank Sum < −8.0.” ANNOVAR software was applied for functional annotation of the detected gene variants. The genotyping information of SNPs was extracted from the above comparison results and variant results. When the coverage depth of a sample for an SNP is <5X, the locus is treated as deletion, which is indicated as NA in the table; when the genotypic mutation frequency of a sample at an SNP is ≥ 0.8 or ≤0.2, the purity of locus is confirmed; if the mutation frequency is between 0.2–0.8 and each allele in the heterozygous genotype should be supported by at least four reads, the SNP is heterozygous mutation. Otherwise, the locus was treated as deletion and expressed as NA in the table; Finally, the genotype results of samples at all SNPs were summarized.

### ED method correlation results

Euclidean Distance (ED) algorithm was used to find markers with significant differences between mixed pools and evaluate the regions associated with traits. The calculation formula of the ED method is as follows:

$$ED=\sqrt{{\left(A_{mut}-A_{wt}\right)}^{2}+{\left(C_{mut}+C_{wt}\right)}^{2}+{\left(G_{mut}+G_{wt}\right)}^{2}+{\left(T_{mut}+T_{wt}\right)}^{2}}$$

Note: A_mut_ is the frequency of A base in the mutant pool, A_wt_ is the frequency of A base in the wild-type pool. The C_mut_ is the frequency of the C base in the mutant pool, and Cwt is the frequency of the C base in the wild-type pool. G_mut_ is the frequency of the G base in the mutant pool, and G_wt_ is the frequency of the G base in the wild-type pool. T_mut_ is the frequency of the T base in the mutant pool, and T_wt_ is the frequency of the T base in the wild-type pool.

In the process of ED analysis, the MMAPPR software package will process SNP as follows.

(1) Filter out SNP of non-secondary allele;
(2) If the frequency of A, T, C, and G in the wild-type mixing pool is greater than or equal to 95%, it will be filtered;
(3) SNPs with sequencing depth lower than 10X in wild-type or mutant pools were filtered.

In the study, the fourth power of the original ED was taken as the correlation value to achieve the function of eliminating background noise, and then local polynomial regression fitting was performed to obtain the fitting curve. Median+3SD of the fitted values of all sites was used as the association threshold for analysis. The associated region is determined based on the association threshold. Candidate SNPs were selected from the associated regions, namely, those with mutation frequency >0.75 and Euclidean distance >0.5. For the candidate SNPs, the annotation results of ANNOVAR were extracted.

### GO enrichment analysis

The GO function of candidate genes was analyzed for significance enrichment. The candidate genes were mapped to each term of GO database (http://www.geneontology.org/) and the gene numbers of each term were calculated to obtain the list and the statistic number of a certain GO functional gene. Hypergeometric tests were then applied to find GO entries that were significantly enriched in genes compared to the whole genomic background.

### KEGG enrichment analysis

KEGG enrichment analysis was carried out based on the hypergeometric distribution. After correction for multiple tests, pathways with Q value ≤ 0.05 were defined as those significantly enriched in candidate genes. The calculation formula is as follows:

$$P=1-\overset{m-1}{\underset{I=0}{\sum }}\frac{\left(\begin{array}{l} M\\ i \end{array}\right)\left(\begin{array}{l} N-M\\ n-i \end{array}\right)}{\left(\begin{array}{l} N\\ i \end{array}\right)}$$

Note: “N” is the number of genes with Pathway annotation in the whole genome; “n” is the number of candidate genes screened according to the selection signal index; “M” is the number of genes annotated as a specific Pathway among all genes; “m” is the number of candidate genes annotated for a specific Pathway.

### SNPs analysis of genes of melatonin synthetic enzymes

PCR amplification was performed in the samples with primers and the results were shown in Table 1. Simply, the reaction mixture was comprised of Premix Taq (TaKaRa Taq Version 2.0 Plus Dye) 25 μL, genomic DNA (20 ng/μL) 1 μL, primer 1 (20 μm) 1 μL, primer 2 (20 μm) 1 μL and with the addition of sterilized water up to total 50 μL. The PCR procedure consisted of a pre-denaturation stage of 95°C for 5 min, 30 cycles of 94°C for 30 s, 60°C for 30 s, and 72°C for 10 s. The final extension period was 72°C for 5 min, followed by cooling to 4°C. PCR products were sent to Jin Weizhi Biotechnology Co., Ltd. for sequencing. Sequence results were compared with DNASTAR software (version 7.1), and SNPs were screened using SeqMan Pro software.

**TABLE 1:** Sequence list of validation primers.

No.	Gene	Site	Upstream primers (5′-3′)	Downstream primers (5′-3′)
P01	MTNRA	29146330	GAA​ATT​GAA​GGC​TTC​TTA​GTT​GGT	GAA​ATT​GAA​GGC​TTC​TTA​GTT​GGT
P02	MTNR1B	1407405	TCA​ACC​TAA​TTT​TGG​GTT​CAA​GAC	TCA​ACC​TAA​TTT​TGG​GTT​CAA​GAC
P03	MTNR1B	1389143	ATT​CTC​TCC​CAT​CAT​TTC​CTG​AGT	GTC​CTG​CTG​CCC​AAC​TTC​TT
P04	MTNR1B	1389193	ATT​CTC​TCC​CAT​CAT​TTC​CTG​AGT	GTC​CTG​CTG​CCC​AAC​TTC​TT
P05	MTNR1B	1388843	ATT​CTC​TCC​CAT​CAT​TTC​CTG​AGT	GTC​CTG​CTG​CCC​AAC​TTC​TT
P06	ASMT	147316	CTC​TCC​CCC​AGC​CTA​TGT​G	CAC​TCA​ATA​TAG​TGT​GCC​TGT​GTG
P07	ASMT	147379	CTC​TCC​CCC​AGC​CTA​TGT​G	CAC​TCA​ATA​TAG​TGT​GCC​TGT​GTG
P08	AANAT	54512530	CCT​CTC​GCT​CAA​TCT​CAA​ACA​C	TCC​TAG​AAT​TTG​AGA​GCA​GGA​GTC
P09	AANAT	54511456	GCC​TTT​TCT​TTA​TTT​CAC​CCA​TTC	CCC​TAA​GAA​CTG​CAC​ATC​AAC​AG

Note: Site P03, P04, and P05 share a pair of primers; Locus P06 and P07 shared a pair of primers.

### Data analysis

Pearson correlation coefficient test between melatonin level and mutation sites was performed using SPSS 20.0 (IBM SPSS Statistics, Armonk, NY, United States) statistical software. *p* < 0.05 was considered statistically significant.

## Results

### The distribution of different levels of melatonin in the tested dairy goats

The distribution of different levels of melatonin in milk and blood samples of 103 dairy goats were shown in Supplementary Figure S1. The goats having melatonin level of 0–0.5 ng/mL in milk (34.95%, 36 goats) was classified as low melatonin group, goats having melatonin level excessive of 1 ng/mL (15.53%, 16 goats) was classified as high melatonin group. The same criteria was used to classify the blood melatonin levels in which, low melatonin group accounted for 44.66% (46 goats) and high melatonin group accounted for 30.10% (31 goats). In general, the dairy goats with melatonin level excessive 1 ng/mL was less than other groups. Based on the results, three dairy goats with the relatively high and three with the relatively low melatonin levels in their milk were selected for whole genome sequencing analysis. The detailed information on these dairy goats were listed in Table 2.

**TABLE 2:** The melatonin levels of selected goats for whole genome sequencing analysis.

Selected goats	Milk (ng/mL)	Blood (ng/mL)
A1-1	2.05	1.04
A1-2	2.09	0.80
A1-3	3.53	0.70
A2-1	0.67	0.26
A2-2	0.55	0.52
A2-3	0.72	0.47

### The statistics of quality control of the original sequencing data

Whole-genome resequencing was performed on blood samples of six goats, and the original Data (PF) was shown in Table 3. Clean Data was obtained by removing low-quality sequences from the original data, as shown in Table 4. The purity of Clean Data after QC excessed more than 99% in PF data, as shown in Table 5. The Clean Data was aligned with the reference Genome sequence by Dragen Genome Pipline and the ratio of alignment matched 99.64%. The number of unique RESDs in the reference Genome accounted for 80.77% of all the reference genomes in the alignment, as shown in Table 6. The average base Quality of Reads range from 35 to 37 Supplementary Figure S2. The average coverage reached 96.05% and the average coverage depth reached 28.32%, as shown in Supplementary Figure S3. The results indicated high quality of the original sequencing data and these data were suitable for subsequent related bioinformatics analysis.

**TABLE 3:** PF Data statistics.

Sample	Length (bp)	Reads	Bases	Q20 (%)	Q30 (%)	GC (%)	N (ppm)
A1-1	150.00	667862868	100179430200	96.99	92.07	43.27	30.02
A1-2	150.00	681108338	102166250700	97.00	92.08	43.51	29.67
A1-3	150.00	637124786	95568717900	96.83	91.77	44.39	29.83
A2-1	150.00	664092002	99613800300	96.85	91.76	43.94	29.77
A2-2	150.00	671900376	100785056400	96.53	91.51	49.58	29.65
A2-3	150.00	683104218	102465632700	96.87	91.93	45.98	29.74

Note: 1) Sample: Name of the sequencing sample. 2) Length: Average length of reads. 3) Reads: Number of sequenced reads. 4) Bases: Total number of bases. 5) Q20 (%): The percentage of bases with Phred values greater than 20 in the total base population. 6) Q30 (%): The percentage of bases with Phred values greater than 30 in the total base population. 7) GC (%): The total number of bases G and C as a percentage of the total number of bases. 8) N (ppm): The number of bases N per million that cannot be determined by sequencing.

**TABLE 4:** Clean Data statistics.

Sample	Length (bp)	Reads	Bases	Q20 (%)	Q30 (%)	GC (%)	N (ppm)
A1-1	149.24	665928370	99384461855	97.52	93.16	43.37	7.26
A1-2	149.26	679194914	101373516161	97.52	93.14	43.60	7.13
A1-3	149.24	635147828	94791990666	97.42	92.97	44.50	7.21
A2-1	149.25	662201660	98831154378	97.42	92.93	43.95	7.26
A2-2	149.21	668759476	99788340330	97.16	92.66	49.57	7.21
A2-3	149.26	680935304	101636103339	97.40	92.95	45.98	7.26

Note: 1) Sample: Name of the sequencing sample. 2) Length: Average length of reads. 3) Reads: Number of sequenced reads. 4) Bases: Total number of bases. 5) Q20 (%): The percentage of bases with Phred values greater than 20 in the total base population. 6) Q30 (%): The percentage of bases with Phred values greater than 30 in the total base population. 7) GC (%): The total number of bases G and C as a percentage of the total number of bases. 8) N (ppm): The number of bases N per million that cannot be determined by sequencing.

**TABLE 5:** Clean Data Ratio statistics.

Sample	PF Reads	Clean Reads	Ratio of Reads (%)	PF bases	Clean bases	Ratio of bases (%)
A1-1	667862868	665928370	99.71	100179430200	99384461855	99.21
A1-2	681108338	679194914	99.72	102166250700	101373516161	99.22
A1-3	637124786	635147828	99.69	95568717900	94791990666	99.19
A2-1	664092002	662201660	99.72	99613800300	98831154378	99.21
A2-2	671900376	668759476	99.53	100785056400	99788340330	99.01
A2-3	683104218	680935304	99.68	102465632700	101636103339	99.19

Note: 1) Sample: Name of the sequencing sample. 2) PF Reads: Number of raw data reads. 3) Clean Reads: Number of data reads after QC. 4) Ratio of Reads (%): Percentage of Clean Reads in the number of PF reads. 5) PF Bases: The number of bases in the original data. 6) Clean Bases: Number of bases after QC. 7) Ratio of Bases (%): Clean Bases Percentage of the number of PF bases.

**TABLE 6:** Sequencing comparison statistics.

Sample	Total Reads	Reads mapped to genome	Mapped Reads Ratio (%)	Uniq Reads	Uniq Reads Ratio (%)
A1-1	665928370	664188270	99.74	533786525	80.16
A1-2	679194914	677590128	99.76	551091460	81.14
A1-3	635147828	633502104	99.74	512685155	80.72
A2-1	662201660	660501371	99.74	539196720	81.42
A2-2	668759476	663020030	99.14	531024333	79.40
A2-3	680935304	679112849	99.73	556727432	81.76

Note: 1) Sample: Name of the sequencing sample. 2) Total Reads: The number of all sequenced reads. 3) Mapped Reads: Number of reads of reference genome on all alignments. 4) Mapped Reads Ratio (%): Ratio of reads to the reference genome. 5) Uniq Reads: The number of reads aligned to a unique position in the reference genome. 6) Uniq Reads Ratio (%): Uniq reads as percentage of all mapped reads. 7) Mean depth: The average sequencing depth of the bases covered.

### Genome-wide SNP detection and annotation

**FIGURE 1:**
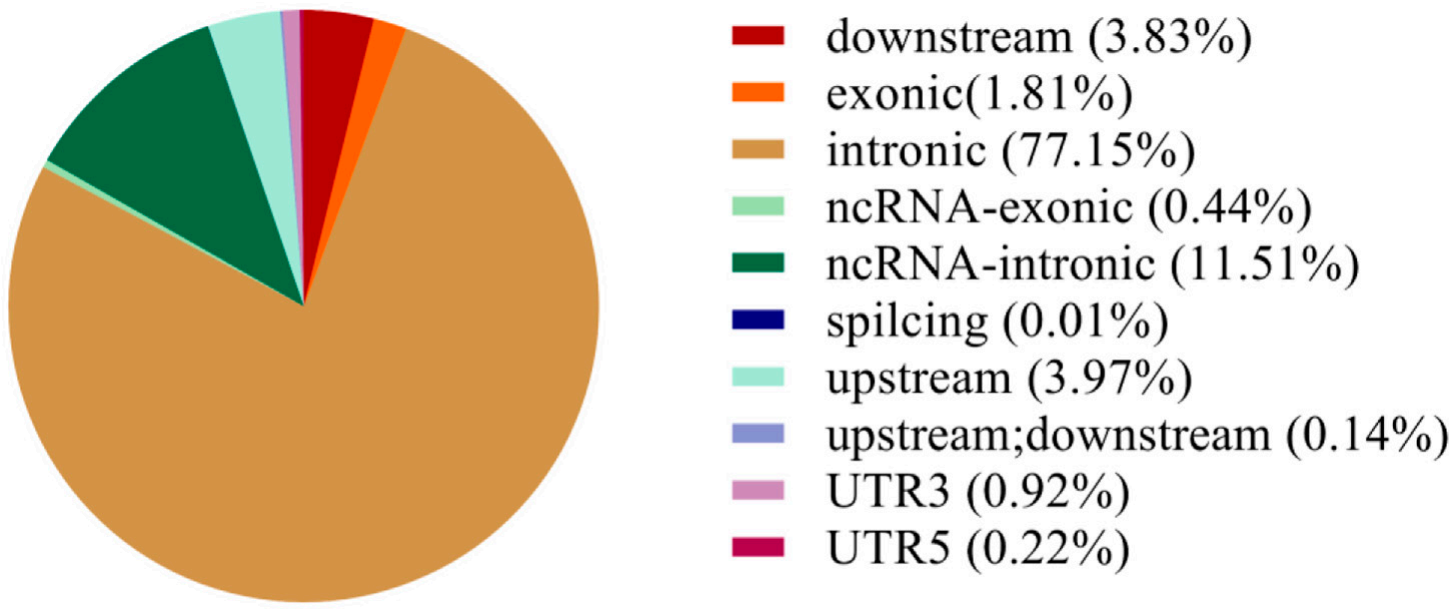
SNP annotation statistics.

Based on the results from comparison, SNPs were detected by Dragen Genome Pipline. After identification of the functional changes caused by mutation sites, SNPs were divided into synonymous and non-synonymous mutations. The results showed that the proportion of non-synonymous mutations was higher than that of synonymous mutations (Table 7). Based on the base complementary pairing principle, SNPs mutation modes were divided into six categories and the focus was given to C:G > T:A and A:T > C:G, as shown in Supplementary Figure S4. The site deletion and mutation of each sample were calculated and the statistical results were shown in Table 8. After screening and filtering the SNPs in the samples, a total of 5,534,640 SNPs were obtained. The results showed that mutation sites were mainly located in the Intronic and ncRNA regions of the genome, and few were located in the UTR5 and UTR3 regions (Figure 1).

**TABLE 7:** Genome-wide SNP type statistics.

Sample	SNP	Non-synonymous	Synonymous	Stop gain	Stop lost
A1-1	10001447	5865	4544	89	11
A1-2	10043386	5884	4485	95	12
A1-3	10223155	6187	4740	94	15
A2-1	9947517	6003	4611	91	10
A2-2	10008310	5656	4310	98	15
A2-3	10101738	5902	4406	88	15

Note: 1) Sample: Name of the sequencing sample. 2) SNP: The total number of SNP. 3) Non-Synonymous: Total number of non-synonymous mutations. 4) Synonymous: Total number of synonymous mutations. 5) Stop gain: A mutation is the number of stop codons (a mutation causes a gene to acquire a stop codon). 6) Stop lost: The number of stop codon deletions (mutations that cause a gene to lose its stop codon).

**TABLE 8:** Sample statistics.

Sample	NA_number	NA_rate (%)	Hetalt_number	Hetalt_rate (%)	Hom_alt_number	Homalt_rate (%)	Ref_number	Ref_rate (%)
A1	875046	15.81	2741602	58.84	858893	18.43	1059099	22.73
A2	1050914	18.99	2770437	61.79	858461	19.15	854828	19.07

Note: Sample: Name of the sequencing sample. NA_number: Number of deletion sites. NA_rate: Ratio of the number of missing sites to the total number of sites. Het_alt_number: Number of heterozygous sites. Het_alt_rate: Ratio of the number of heterozygous sites to the total number of non-deletion sites. Hom_alt_number: The number of homozygous sites. Hom_alt_rate: Ratio of the number of homozygous loci to the total number of non-deletion loci. Ref_number: Reference consensus number. Ref_rate: The ratio of the number of reference consensus sites to the total number of non-deletion sites.

## Results of ED method association

**FIGURE 2:**
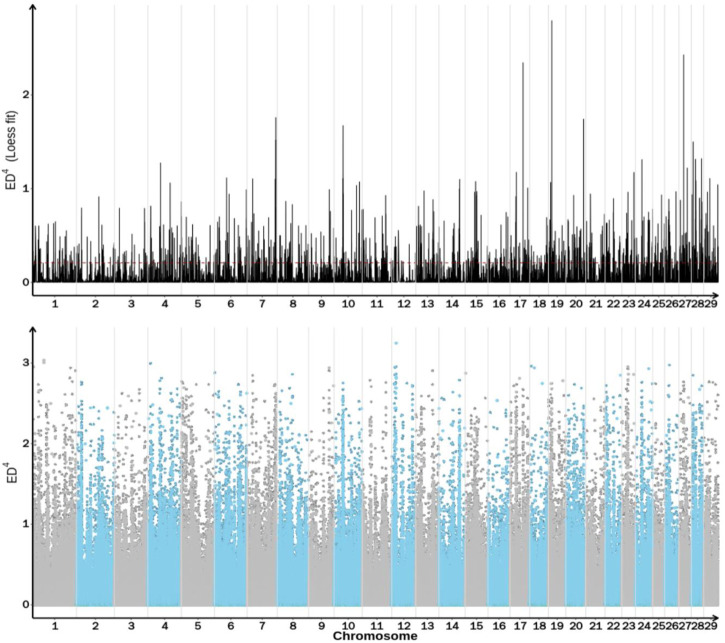
Distribution of ED association values across the genome.

Association analysis of 5,534,640 SNPs was performed using ED analysis and 2,740,786 SNPs were selected for further analysis (Table 9). The distribution of these SNPs on each chromosome is shown in Figure 2. The association threshold was set up as Median +3SD of all selected sites and the calculated median+3SD value was 0.2086. Based on the association threshold, a total of 1,095 regions with a total length of 34,387,035 bp were obtained, as shown in Supplementary Table S1. Candidate SNPs were selected from the associated regions with mutation frequency >0.75 and Euclidean distance >0.5. A total of 34,921 SNPs were identified, ANNOVAR annotation analysis showed that these selected candidate SNPs involved in 1,177 genes.

**TABLE 9:** SNP site filtering statistics.

Total	Biallelic	Frequency	NA
5,534,640	4,011,834	3,331,668	2,740,786

Note: Total numbe: Total number of SNPs. Biallelic: The number of SNPs after filtering non-secondary alleles. Frequency: The number of SNPs after the frequency of filtering bases is greater. Than or equal to 95%. NA: The number of SNPs after sequencing depth of wild-type or mutant pools was lower than 10X.

The abscissa is the chromosome name, and the dot plot represents the ED value of each SNP locus. The line graph shows the ED value after fitting. The higher the ED value, the better the correlation effect of the point, and the blue shadow represents the interval to be located.

### GO enrichment analysis

**FIGURE 3:**
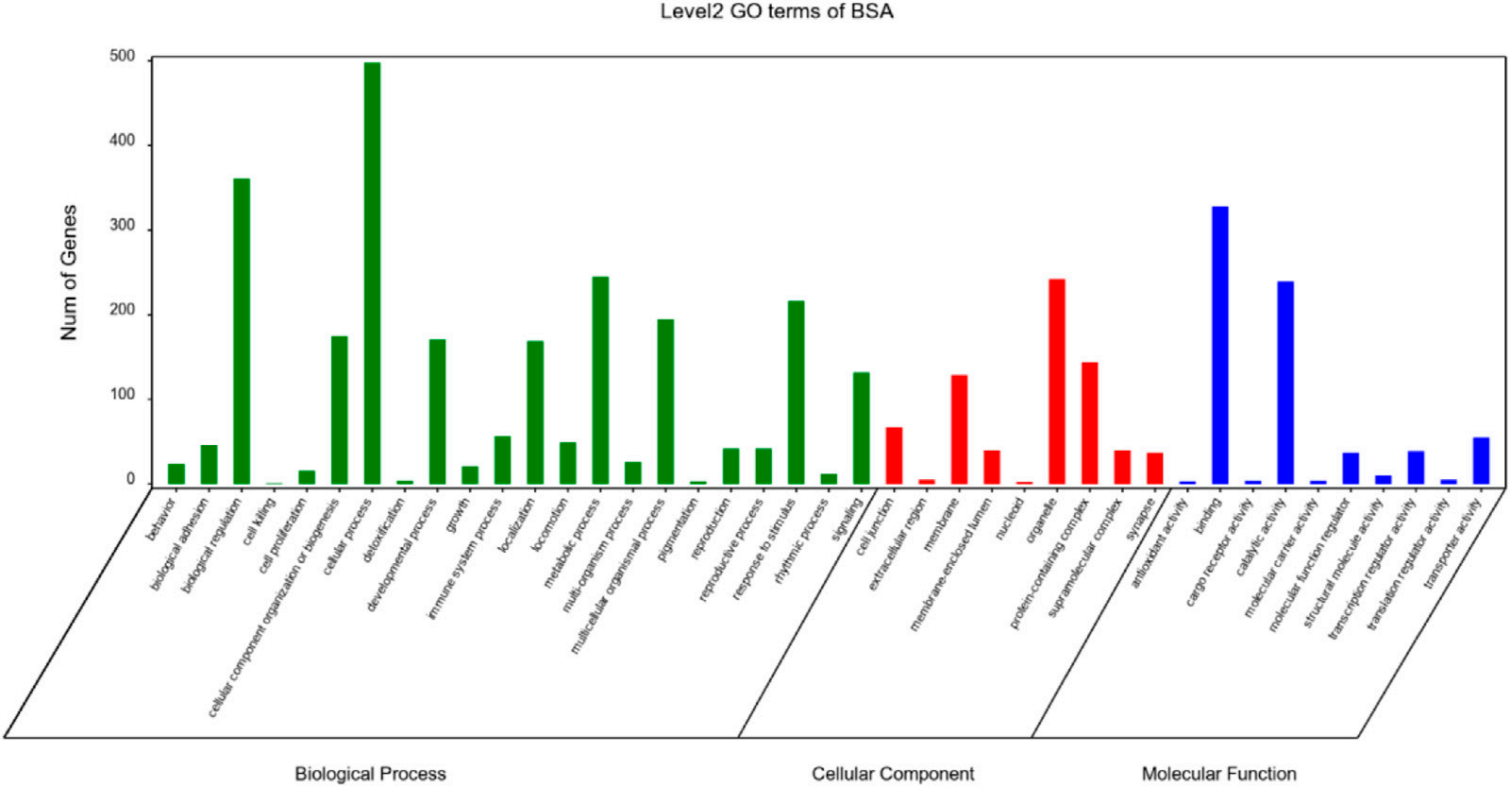
GO functional classification statistics.

After annotation for the GO function of candidate genes, it was found that these genes were enriched into Molecular Function, Cellular Component and Biological Process, respectively, as shown in Figure 3. A total of 10 terms enriched to Molecular Function and 328 genes enriched to Binging. A total of 9 terms were enriched to Cellular Component and 242 genes were enriched to Organelle.

**FIGURE 4:**
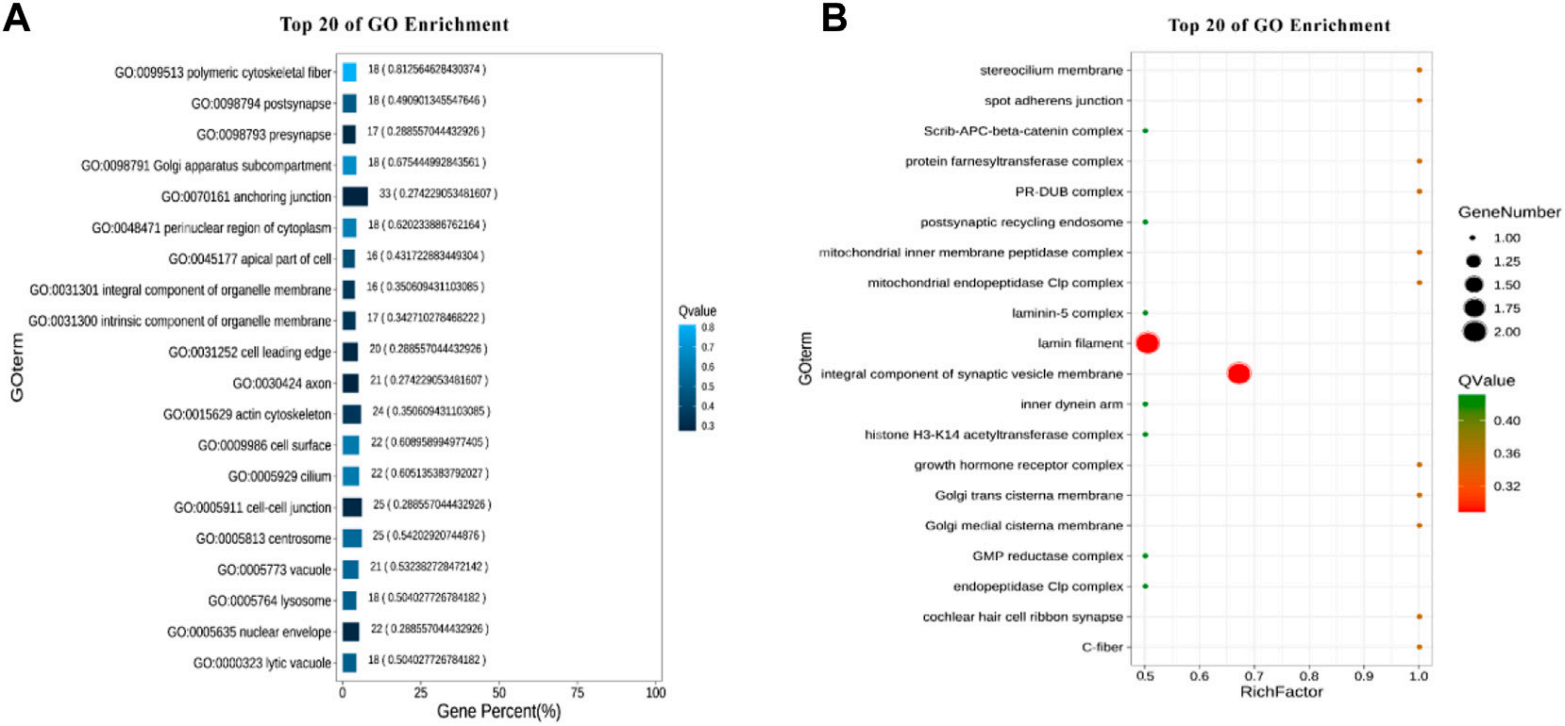
GO enrichment results of cellular component **(A)** Enrichment bar chart. Note: The ordinate is GO term and the abscissa is the percentage of all differences accounted for by this GO term. From the largest to the smallest, the top 20 are selected. The darker the color, the smaller the Q value. The number of GO term and Q value are labeled above. **(B)** Bubble chart. Note: The ordinate is GO term, and the abscissa is enrichment factor (the number of differences in the GO term divided by all the numbers). From the largest to the smallest, the top 20 are selected. Size of bubble area: the number of genes belonging to this GO in the target gene set; Bubble color: enrichment significance, that is, the size of Q value; The size is the quantity, and the redder the color, the smaller the Q.

**FIGURE 5:**
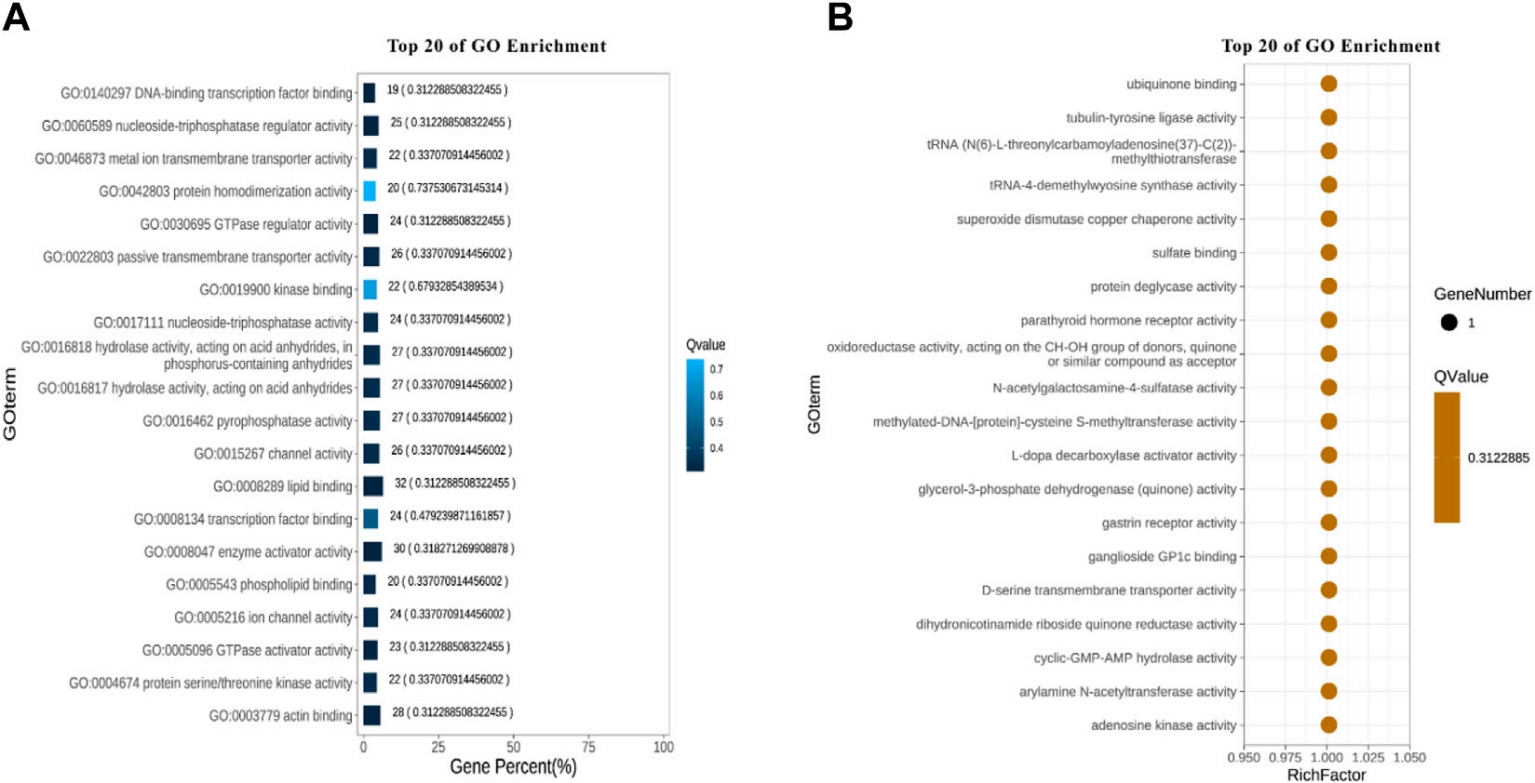
Molecular functional GO enrichment results **(A)** Enrichment bar chart. **(B)** Bubble chart.

**FIGURE 6:**
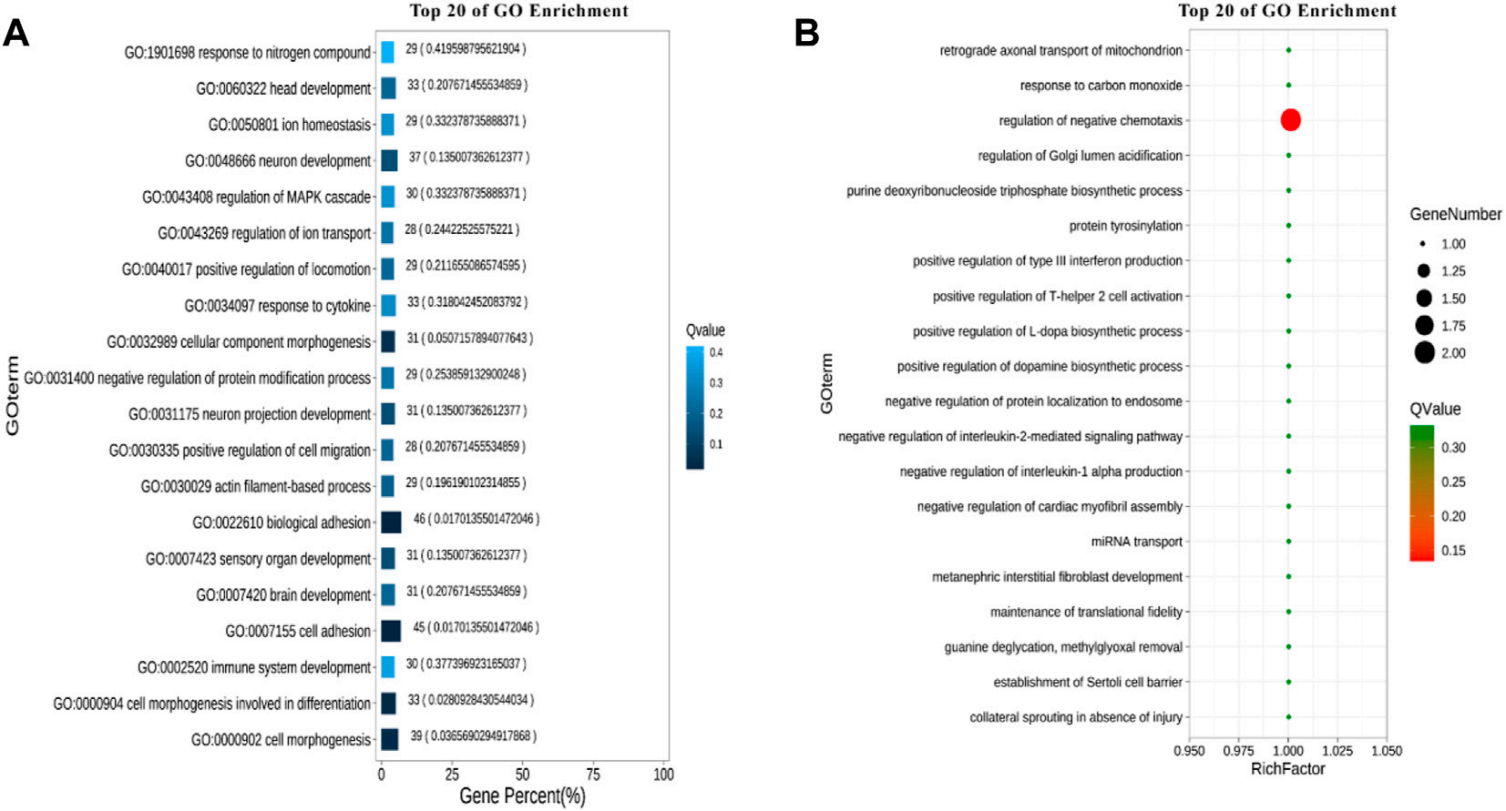
GO enrichment results of biological processes. **(A)** Enrichment bar chart. **(B)** Bubble chart.

A total of 22 terms were enriched in Biological Process and 498 genes were enriched in cellular process. To identify significantly enriched GO items, the GO function of candidate genes was analyzed for significance enrichment with hypergeometric test. The results indicated that the anchoring junction was the most enriched differential genes in Cellular Component (Figure 4). In Molecular Function, only one gene was found in the TOP 20 pathways and the Rich Factor is 1 (Figure 5). Among them the lipid binding pathway represented the largest amount of all differentiated GO term. The pathway with the highest concentration of differential genes in Biological Process is the regulation of negative chemotaxis (Figure 6). Among them, biological adhesion pathway accounted for the largest percentage of the GO term in all differentiated genes.

### KEGG enrichment analysis

**FIGURE 7:**
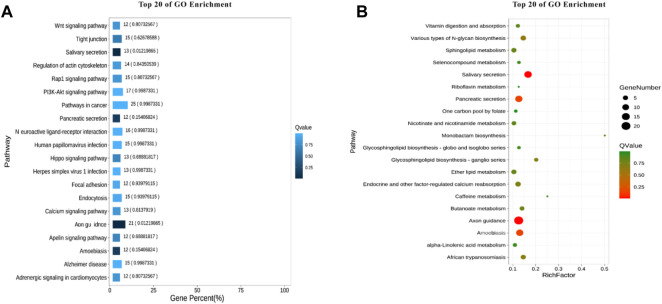
KEGG enrichment analysis. **(A)** Enrichment bar chart. **(B)** Bubble chart.

In organisms, different genes coordinate with each other to perform biological functions. For example, the different genes can share the same pathway to produce same effect. Pathway analysis is helpful to further understand the function of genes. Based on the principle of hypergeometric distribution, the genes of the whole genome were served as background genes, and the candidate genes were analyzed by KO, as shown in Figure 7. The pathways with the highest number of enriched genes are Pathways in cancer. The Pathways accounted for the largest percentage of all different pathways were Axon guidance.

### Correlation analysis of SNPs of *AANAT*/*ASMT* and *MT1*/*MT2* non-synonymous mutations

**FIGURE 8:**
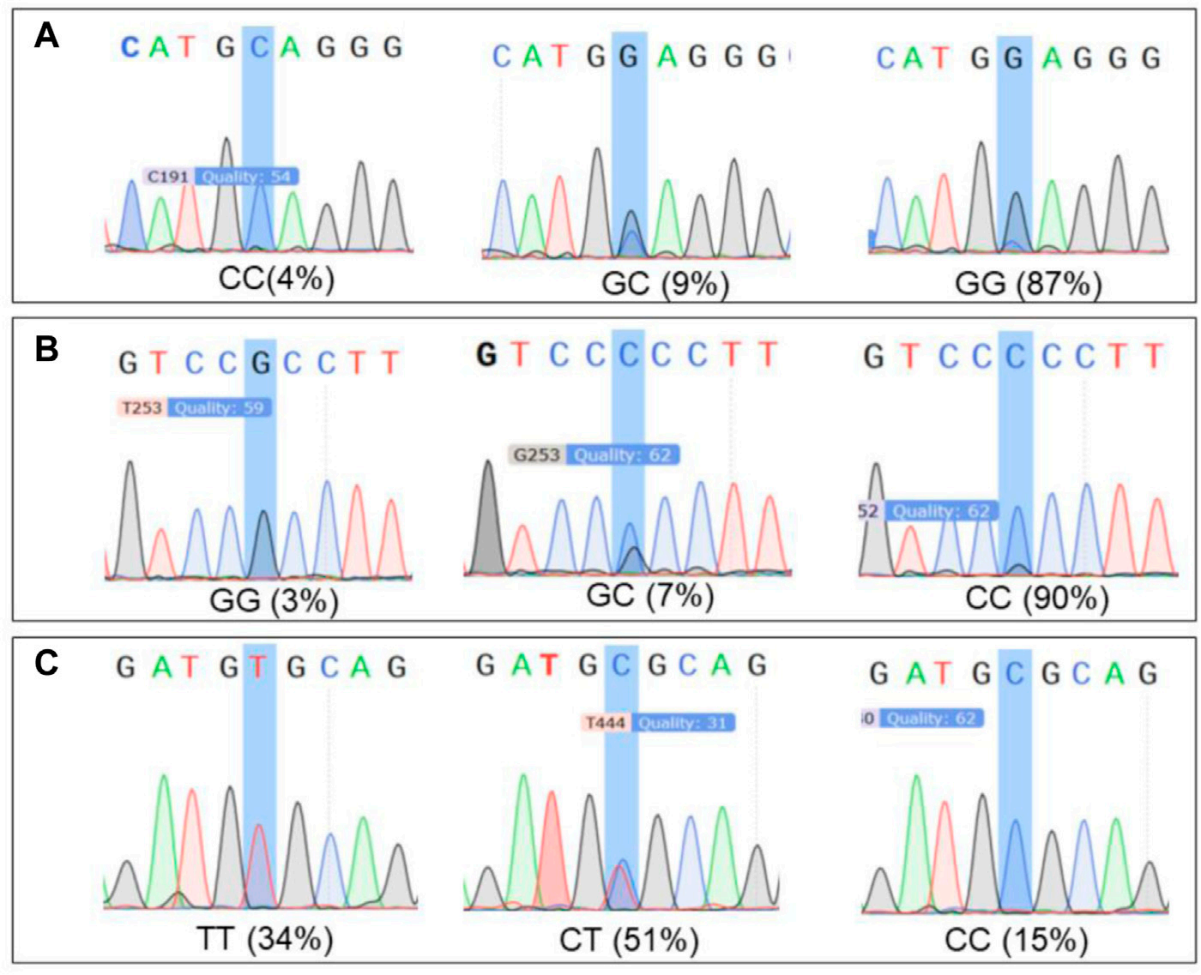
The proportion of significant locus genotypes. Note: **(A)** 147316 loci genotypes. **(B)** 147379 loci genotypes. **(C)** 1389193 loci genotypes.

SNPs of AANAT/ASMT and MT1/MT2 exon mutations are shown in Table 10. The SNP markers are at position of 147316, 147379 and 1389193, respectively, as shown in Table 11. As shown in Figure 8, the dominant genotype of SNP markers at position of 147316 was GG with the proportion of 87%, while the other genotypes were CC (4%) and GC (9%). The dominant genotype of SNP at position of 147379 was CC with the proportion of 90%, and the other genotypes were GC (7%) and GG (3%). The dominant genotype of SNP marker at position of 1389193 was CT with the proportion of 51%, and the other genotypes were CC (15%) and TT (34%). The melatonin levels of genotype CC at position 147316, GG at position 147379 and CC at position 1389193 were all higher than the average melatonin level of 1.05 ng/mL, as shown in Table 12.

**TABLE 10:** Summary of non-synonymous mutations.

No.	Chromosome	Locus	Pre-mutated base	A mutated base	Gene		Amino acid change
P01	NC_030834.1	29146330	C	A	MTNR1A		A/E
P02	NC_030836.1	1407405	G	T	MTNR1B		F/L
P03	NC_030836.1	1389143	T	C	MTNR1B		K/E
P04	NC_030836.1	1389193	T	C	MTNR1B		H/R
P05	NC_030836.1	1388843	C	T	MTNR1B		G/S
P06	NW_017,189,541.1	147316	G	C	ASMT		E/Q
P07	NW_017,189,541.1	147379	C	G	ASMT		P/A
P08	NC_030826.1	54512530	C	T	AANAT		V/I
P09	NC_030826.1	54511456	T	C	AANAT		Q/R

**TABLE 11:** Significance test for mutation sites.

Gene	AANAT		ASMT		MTNR1A	MTNR1B			
No.	P08	P09	P06	P07	P01	P05	P03	P02	P04
Site	C/T	T/C	G/C	C/G	C/A	C/T	T/C	G/T	T/C
(P)	0.866	0.564	0.001	0.011	0.758	0.860	0.630	0.601	0.001

Note: *p* < 0.05 indicates a significant correlation at the 0.05 level and *p* < 0.01 indicates a significant correlation at the 0.01 level.

**TABLE 12:** Melatonin concentrations at significant loci genotypes.

Site	P06/147316			P07/147379			P04/1389193		
genotype	GG	CC	GC	GG	GC	CC	CT	CC	TT
melatonin	0.87	5.61	0.77	5.27	0.82	0.93	0.64	3.15	0.75

Note: The unit is ng/mL.

## Discussion

Dairy products are important human non-staple food with high nutritional value (Lu and Miller, 2019). Among them, goat milk contains higher protein and fat contents than cow milk and its nutritional value is close to the human milk (Hodgkinson et al., 2018). Even though the numbers of dairy goats have been dramatically expended, it still cannot satisfy the global demanding for the goat milk and its dairy products. To increase production and quality of the goat milk, the first step is to cultivate the dairy goats with the best traits for milk production. To achieve this purpose, the breeding strategy is critical. The traditional breeding strategy is the crossover of the animal with the selected traits. But, for the last decade, the breeding has advanced to the technology of molecular biology (Lenstra et al., 2012). By use of the DNA marker and microarray technologies, the reference populations can be rapidly established with a large scale (Kraus et al., 2015). Compared with other farming animals, the modern technology for dairy goat breeding has lagged behind for a while, but currently, it has also been advanced into the era of microarray breeding (Gipson, 2019). By comparing the high and low fecundity of the dairy goats and sequencing their whole genes, Lai et al. have identified some candidate genes related to reproductive traits including non-synonymous exon SNPs in SETDB2 and CDH26 genes (Lai et al., 2016). In the previous study, we have found that the transgenic dairy goats with overexpression of AANAT and ASMT not only had the improved milk quality, but also had stronger anti-inflammatory ability than that of wild type (Tao et al., 2018; Wu et al., 2022). AANAT and ASMT are rate limiting enzymes for melatonin synthesis and their gene expressions and activities determine endogenous melatonin production. Melatonin is distributed in all cells, tissues and organ. It is synthesized in the mitochondria (He et al., 2016) and majority of its actions may also occur in the level of mitochondria (Reiter et al., 2018). It participates in a variety of biological functions. For example, melatonin regulates the reproductive action in vertebrates and has strong influence on biological rhythmicity of many organisms (Johnston and Skene, 2015; Talpur et al., 2018). Season, light and age all affect the level of melatonin in the body (Adamsson et al., 2017; Cardinali, 2021). Since the overexpression of AANAT and ASMT positively associates with milk production and quality of the goats, we believed that this beneficial effect is mediated by the increased endogenous melatonin production. Therefore, in the study, 103 dairy goats were included to measure their milk and serum melatonin levels. Among them, 6 goats (3 with highest and 3 with lowest melatonin levels) were selected for whole genome screening analysis and correlation analysis of key SNPs of AANAT and ASMT with melatonin level.

A total of 5,534,640 SNPs were obtained by preliminary screening and the mutations mainly occurred in the Intronic and ncRNA regions of the genome. Further ED association analysis was conducted on these SNPs and a total of 34,921 SNPs were selected, involving 1,177 genes. GO enrichment analysis showed that the lipid binding is one of the most significant pathways. The lipid binding pathway involves in genes set related to lipid structure, which is the main component of cell membrane and involves in signal transduction (Fahy et al., 2011). It is well known that melatonin is a small lipid soluble molecule and plays its biological function mainly through the membrane receptor MT1/MT2 (Liu et al., 2016). It appears that the high level of melatonin is required to upregulate the gene expression of this pathway (Dies et al., 2015) and the increased lipid metabolism is directly related to milk quality. In addition, KEGG enrichment analysis indicated that the melatonin also impacted the Axon guidance pathway which is important for signal transduction. The Axon guidance pathway and lipid binding pathway have much functional overlap (Negishi et al., 2005).

As mentioned above, melatonin synthesis depends on the expression and activities of AANAT and ASMT and its biological functions are mediated by MT1 and MT2 receptors. Therefore, we specifically analyzed the SNPs in the exon regions of these four genes (AANAT, ASMT, MT1 and MT2). Since alterations of SNPs in these genes will significantly affect the level of melatonin as well as its functions. Yao et al. (2022) have reported that 3 SNPs in AANAT and 4 SNPs in ASMT were significantly correlated with melatonin levels in blood and milk of Holstein cows. In this study, the similar results were observed in the dairy goats, nine non-synonymous loci located in exon regions of AANAT and ASMT. Pearson correlation coefficient test showed that three out of them were significantly correlated to melatonin production. To determine the genotype, the genotype information of the mutant sites of 103 dairy goats was analyzed. The results showed that the milk and serum melatonin concentrations in CC genotype 147316, GG genotype 147379 and CC genotype 1389193 were all significantly higher than the average melatonin level (1.05 ng/mL) of the tested goats in the study. The data strongly suggests that the mutations of these three SNPs would significantly affect the level of melatonin in dairy goats. As we know that the endogenous melatonin is positively associated with the milk quality and yield of Holstein cows. Elevated melatonin not only increases the protein and fat content but also reduce the somatic cell number of the milk. In addition, milk with high level of melatonin is suitable for elderly consumers for their sleep or other biological rhythmic regulation (Singh et al., 2011). Therefore, these three SNPs can serve as the molecular markers to selectively breed the dairy goats to improve quality and yield of milk. Especially the genotypes of CC 147316 and GG 147379 are the most favorable molecular markers since the dairy goats with these genotypes have five folds of higher melatonin level than the average value (1.05 ng/mL). Based on our best knowledge, this is the first study to identify the association between the SNPs of melatonin synthetic enzymes and endogenous melatonin level, as well as their potential link to the milk quality and yield in the dairy goats. The data strongly suggest that these SNPs can serve as the molecular markers to breed dairy goats with beneficial traits in the large scale.

## Data Availability

Whole genome sequencing data of dairy goats have been successfully submitted to the National Center for Biotechnology Information. SRA data: PRJNA941958.

## Supplementary material

SUPPLEMENTARY FIGURE S1 Distribution of the phenotypes of dairy goats related to their melatonin levels in milk and blood.

SUPPLEMENTARY FIGURE S2 Mean mass distribution of sequencing reads. Note: The abscissa is the average base Quality of Reads. The ordinate is the number of Reads in the sequencing data.

SUPPLEMENTARY FIGURE S3 Sample depth distribution. Note: The abscissa is the coverage depth; The ordinate is the number of bases covered at the specified depth of coverage.

SUPPLEMENTARY FIGURE S4 Cumulative distribution curve of SNP distances. Note: The abscissa is the number of SNP sites with different mutation types. The ordinate is the classification of six mutation types.

SUPPLEMENTARY TABLE S1 Mutation area calculated based on median+3SD.
